# Supplementary material for: Brieflow: an integrated computational pipeline for high-throughput analysis of optical pooled screening data
Source: Nat Commun. 2026 May 30;17:6997. doi: 10.1038/s41467-026-73643-7 (PMC13392126; doi:10.1038/s41467-026-73643-7)
Supplement: Supplementary file 1 — Supplementary Information [file 41467_2026_73643_MOESM1_ESM.pdf]

Supplementary Information

Brieflow: An Integrated Computational Pipeline for High-Throughput Analysis of Optical Pooled Screening Data

Matteo Di Bernardo, Roshan S. Kern, Ana Karla Cepeda Diaz, Alexa Mallar, Samuel J. Choi, Andrew Nutter-Upham, Sebastian Lourido, Paul C. Blainey, Iain Cheeseman

Supplementary Fig. 1

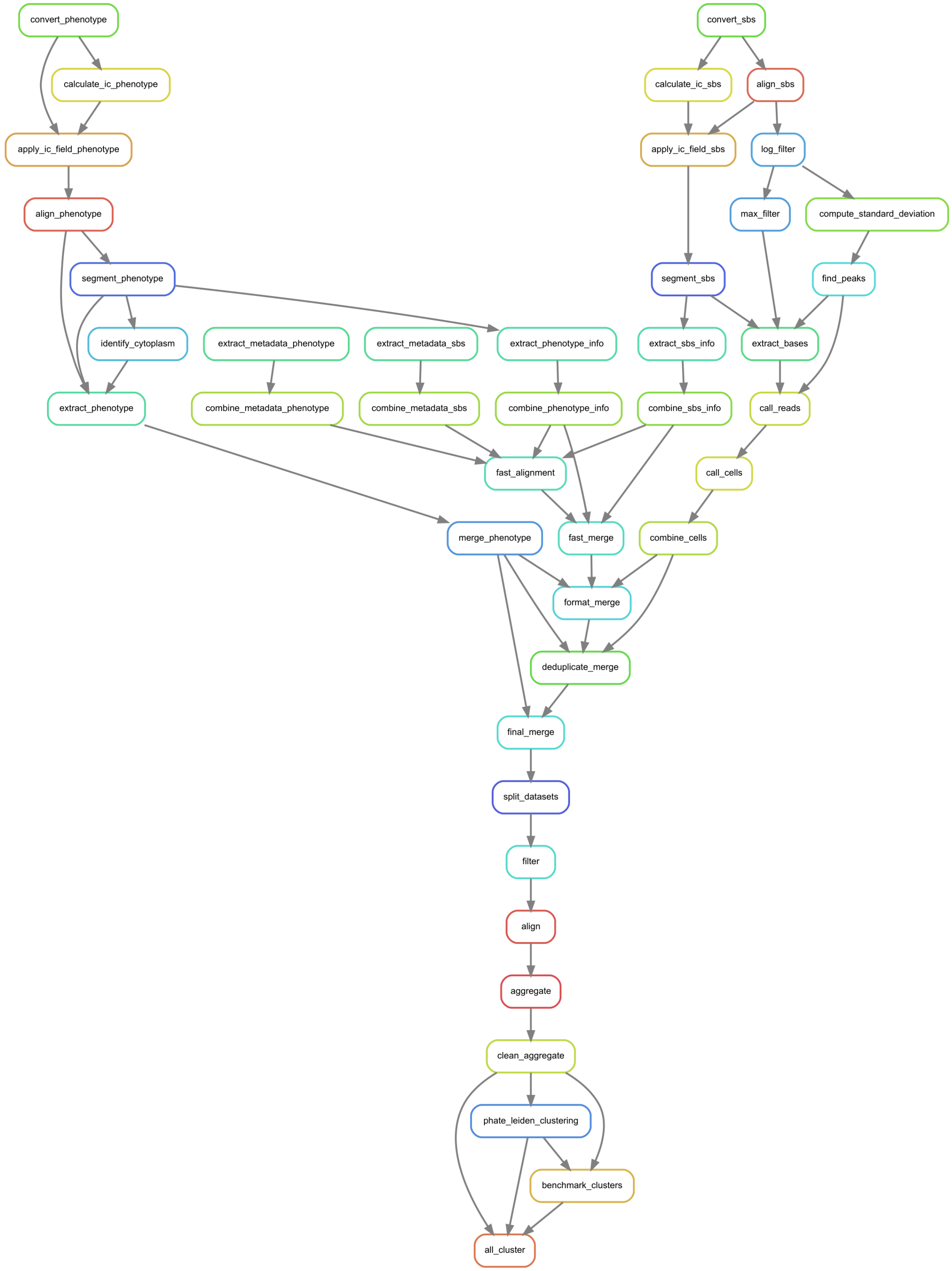

Supplementary Fig. 1: Snakemake-generated rule graph for the Brieflow analysis pipeline, showing the directed acyclic graph of all processing steps from raw data conversion through final clustering and benchmarking. Node border colors are automatically assigned by Snakemake and do not encode module identity.

Supplementary Fig. 2

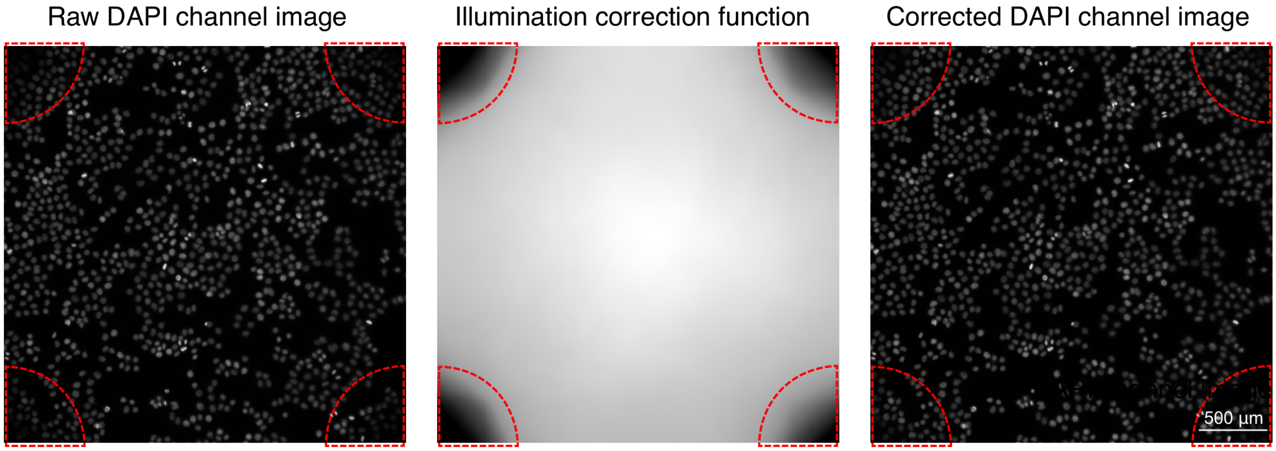

Supplementary Fig. 2: Illumination correction for phenotypic image processing. Raw DAPI channel image (left), the computed illumination correction function (center), and the corrected DAPI channel image (right) for a representative field of view. Red dashed circles highlight well corners where illumination intensity is reduced, demonstrating the correction of vignetting artifacts. Scale bar, 500 µm.

Supplementary Fig. 3

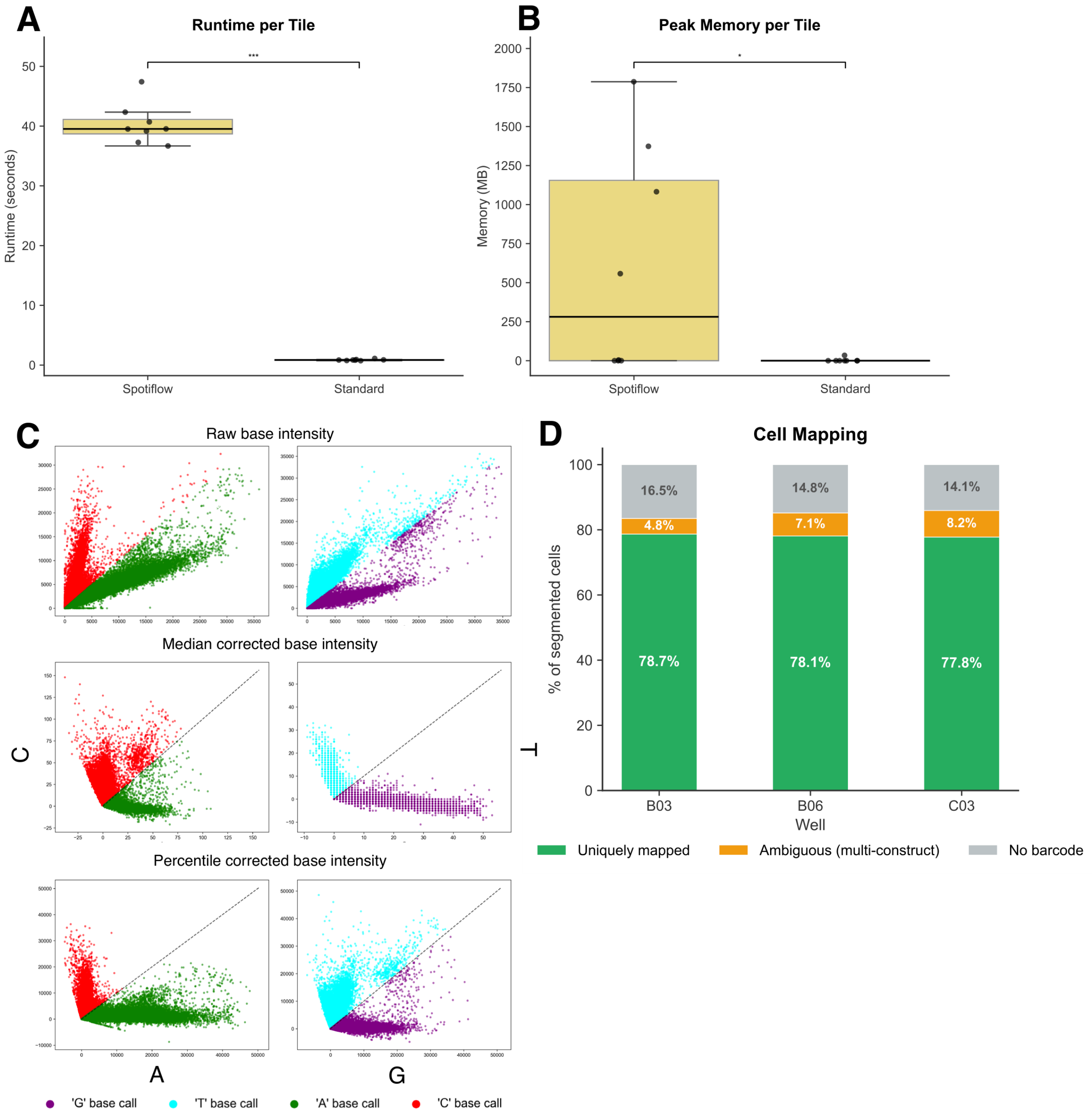

Supplementary Fig. 3: Benchmarking of spot detection methods and normalization strategies. a. Runtime comparison between Spotiflow and the standard method (n = 8 tiles, one per plate), showing substantially longer processing time for Spotiflow (40.3 s vs. 0.9 s per tile). b. Peak memory usage comparison (n = 8 tiles), showing higher memory consumption for Spotiflow (601 MB vs. 4 MB per tile). c. Comparison of normalization strategies for base calling. Raw fluorescence intensities (top row) are transformed using median normalization (middle row) or percentile normalization (bottom row). Each scatter plot shows the fluorescence intensity of one base versus its complementary base (left panels: base A vs. base C; right panels: base T vs. base G). Both approaches improve discrimination between complementary base pairs, with colored points indicating base assignments. d. Validation of the Sequencing-by-Synthesis module on an independent positive-control dataset: a T7-based in situ sequencing experiment with three barcode constructs of known identity, enabling direct assessment of mapping accuracy. Stacked bar plots show the proportion of segmented cells uniquely mapped to a single construct, ambiguously mapped to multiple constructs, or unmapped, across three wells.

Supplementary Fig. 4

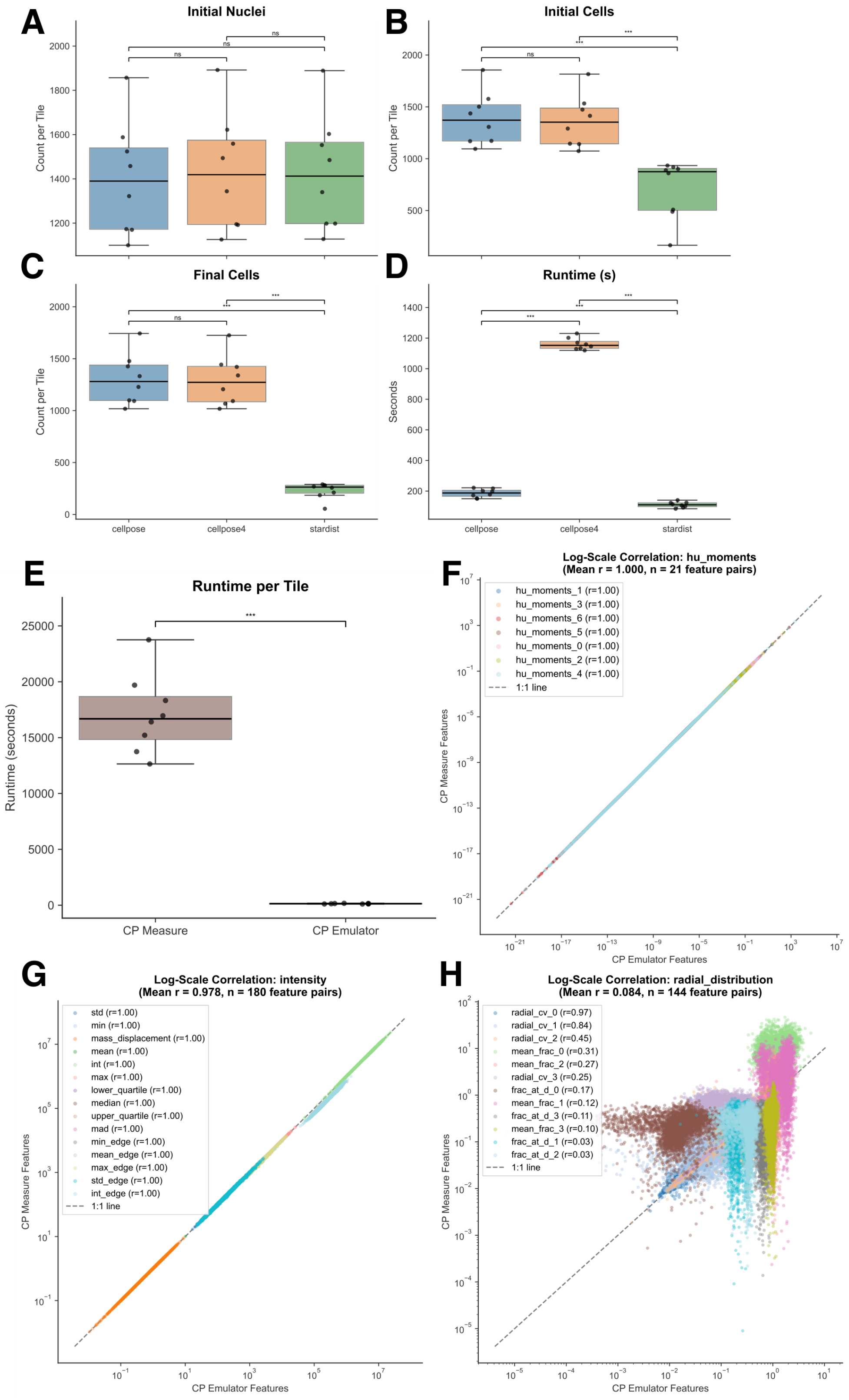

Supplementary Fig. 4: Comparative analysis of segmentation methods and feature extraction implementations. a. Mean initial nuclei detected per tile (n = 8 tiles, one per plate) across Cellpose, Cellpose4, and StarDist, showing similar counts. b. Mean initial cells detected per tile, showing substantially fewer cells detected by StarDist. c. Mean final cells per tile after reconciliation of cell and

nuclear measurements, showing that Cellpose and Cellpose4 retain most cell–nuclei pairs while StarDist shows a large reduction. d. Runtime comparison of segmentation methods, with Cellpose4 substantially slower than Cellpose and StarDist. e. Runtime comparison between cp_measure and the Brieflow emulator for feature extraction (n = 8 tiles, one per plate), showing approximately 125-fold faster processing by the emulator. f. Log-scale correlation of cellular Hu moment features between the Brieflow emulator and cp_measure, showing strong agreement (mean r = 1.000). g. Log-scale correlation of cellular intensity features between implementations, showing high agreement (mean r = 0.978). h. Log-scale correlation of cellular radial distribution features between implementations, showing minimal agreement (mean r = 0.084). This discrepancy reflects differences in the computation of multi-step radial distribution features between implementations. Each dot in f–h represents one cell from 8 benchmark tiles, with colors indicating distinct feature measurements and r denoting the Pearson correlation coefficient.

Supplementary Fig. 5

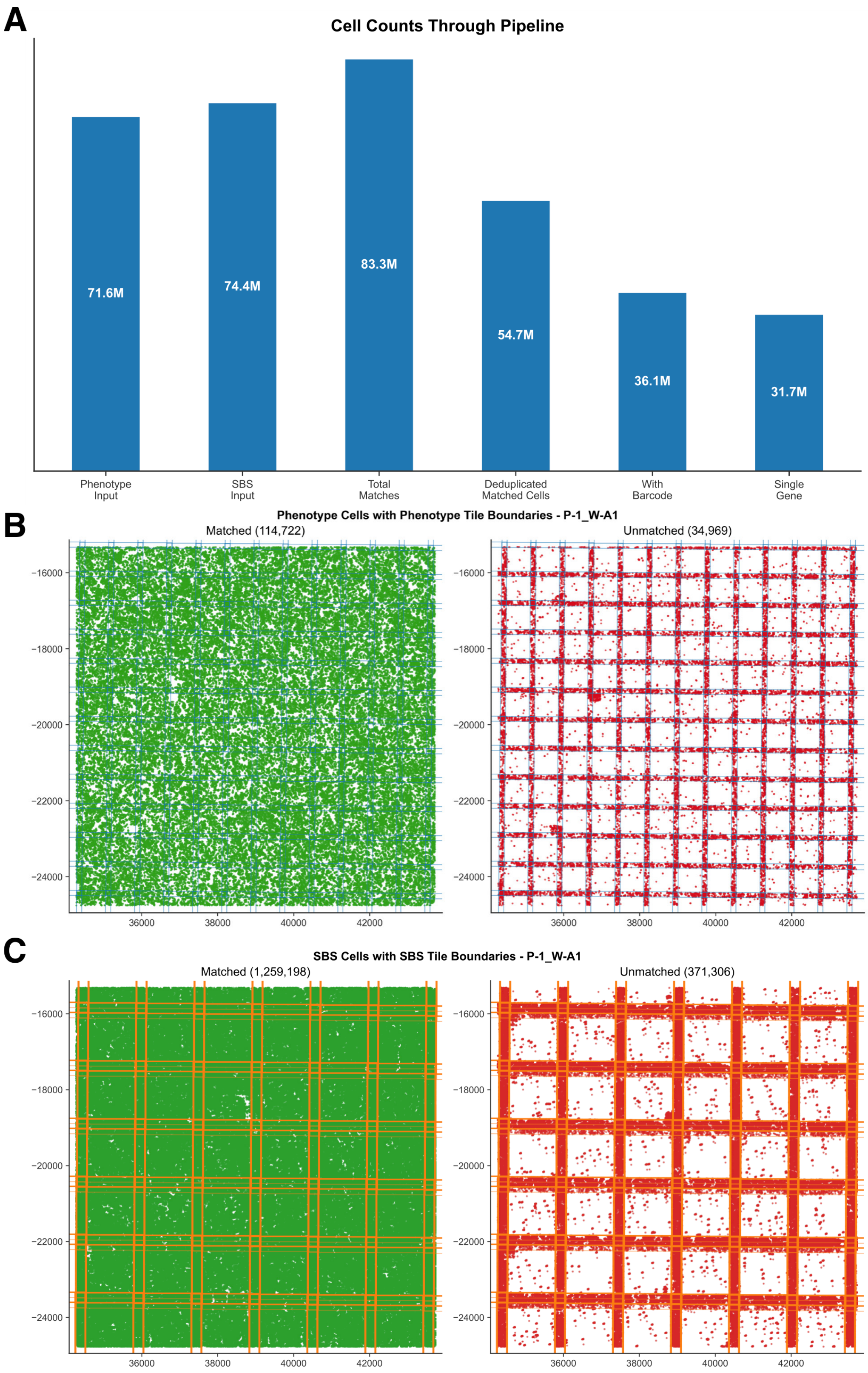

Supplementary Fig. 5: Merge performance and spatial registration validation. a. Cell counts at each stage of the Brieflow pipeline, from Phenotype input (71.6M) and SBS input (74.4M) through total matches (83.3M), deduplicated matched cells (54.7M), cells with barcode (36.1M), to cells with single gene assignment (31.7M). Total matches exceeds individual input counts because overlapping

fields of view in image acquisition cause the same cell to be captured more than once, producing one-to-many matches that are subsequently resolved during deduplication. b. Spatial distribution of matched (left, green) and unmatched (right, red) phenotype cells for a representative well, with phenotype tile boundaries overlaid. Unmatched cells concentrate at tile boundaries where overlapping fields of view produce duplicate detections. c. Spatial distribution of matched (left, green) and unmatched (right, red) SBS cells for the same well, with SBS tile boundaries overlaid, confirming that cell loss during merge reflects boundary deduplication rather than registration failure.

Supplementary Fig. 6

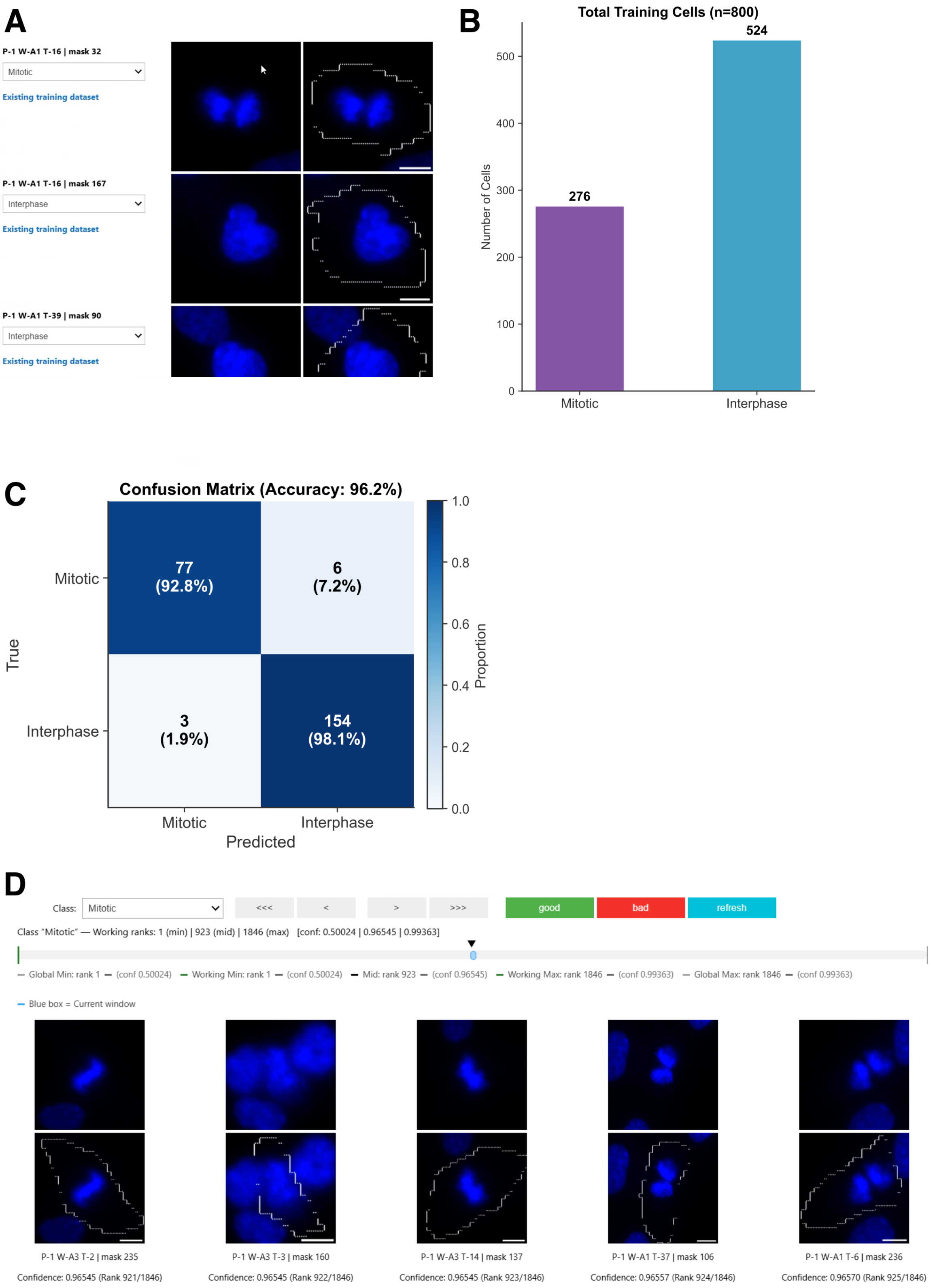

Supplementary Fig. 6: Development and validation of a mitotic cell classifier. a. Interactive labeling interface showing representative cell images across multiple channels, with class assignment dropdown and existing dataset indicators. b. Training dataset composition (n = 800 cells total: 276 mitotic, 524 interphase). c. Confusion matrix on the held-out test set, showing 96.2% overall

accuracy with 92.8% sensitivity for mitotic cells and 98.1% sensitivity for interphase cells. d. Rankline interface (a visualization tool that ranks predictions by model confidence for empirical threshold determination), displaying mitotic cells ranked by prediction confidence with cell images across DAPI and tubulin channels.

Supplementary Fig. 7

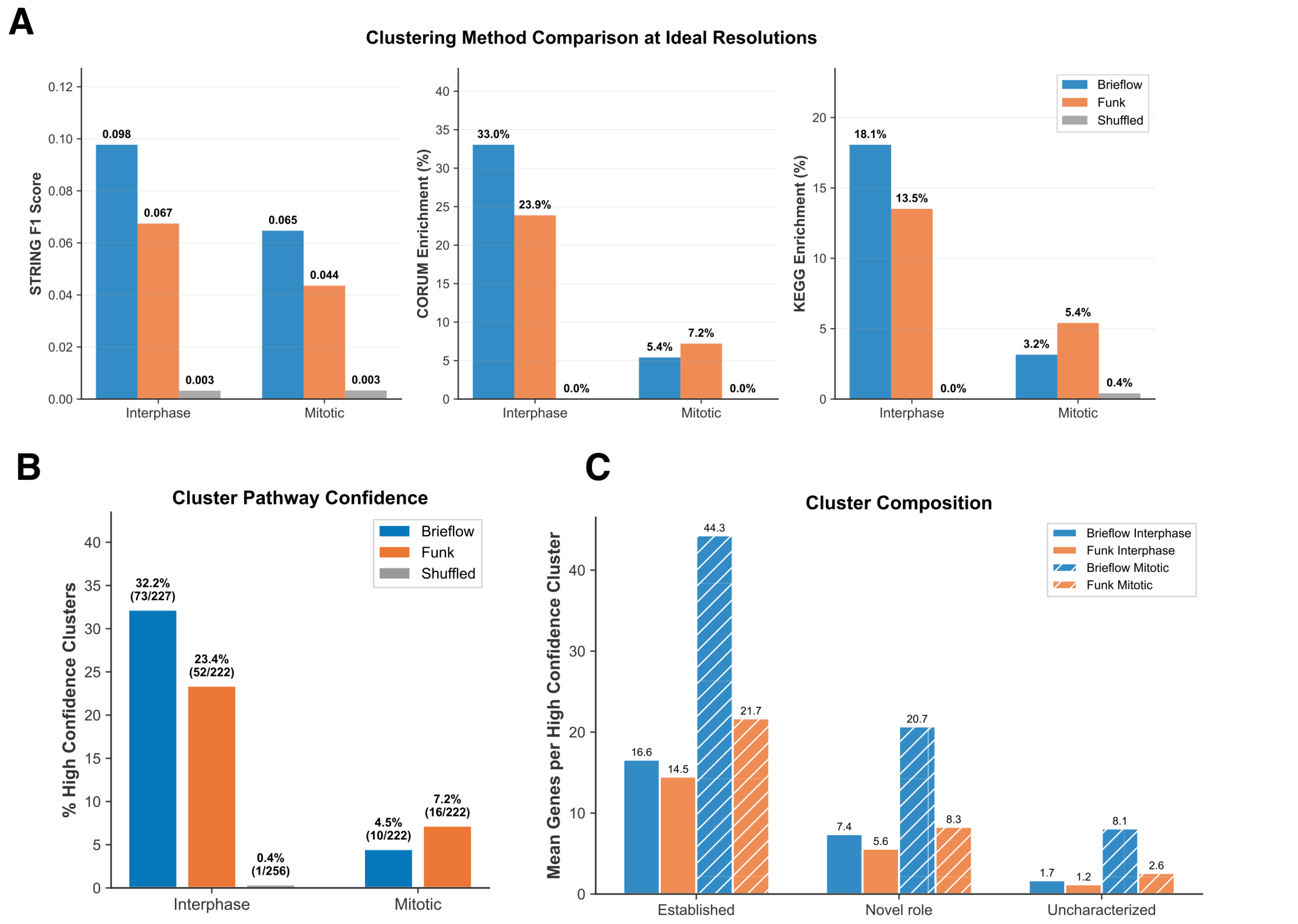

Supplementary Fig. 7: Clustering benchmarks, MozzareLLM validation, and gene classification. a. Comparison of clustering quality metrics between Brieflow, Funk et al., and shuffled controls at their respective optimal resolutions. Left: STRING F1 scores for interphase and mitotic populations. Center: CORUM enrichment. Right: KEGG enrichment. Shuffled controls show near-zero scores across all metrics. b. Percentage of all clusters at the optimal Leiden resolution receiving high-confidence MozzareLLM pathway annotations (absolute counts shown in parentheses) for Brieflow interphase (32.2%), Funk et al. interphase (23.4%), shuffled interphase (0.4%), Brieflow mitotic (4.5%), and Funk et al. mitotic (7.2%). c. Mean number of genes per high-confidence cluster classified as established, novel-role, or uncharacterized, shown for Brieflow and Funk et al. across interphase and mitotic populations.

Supplementary Fig. 8

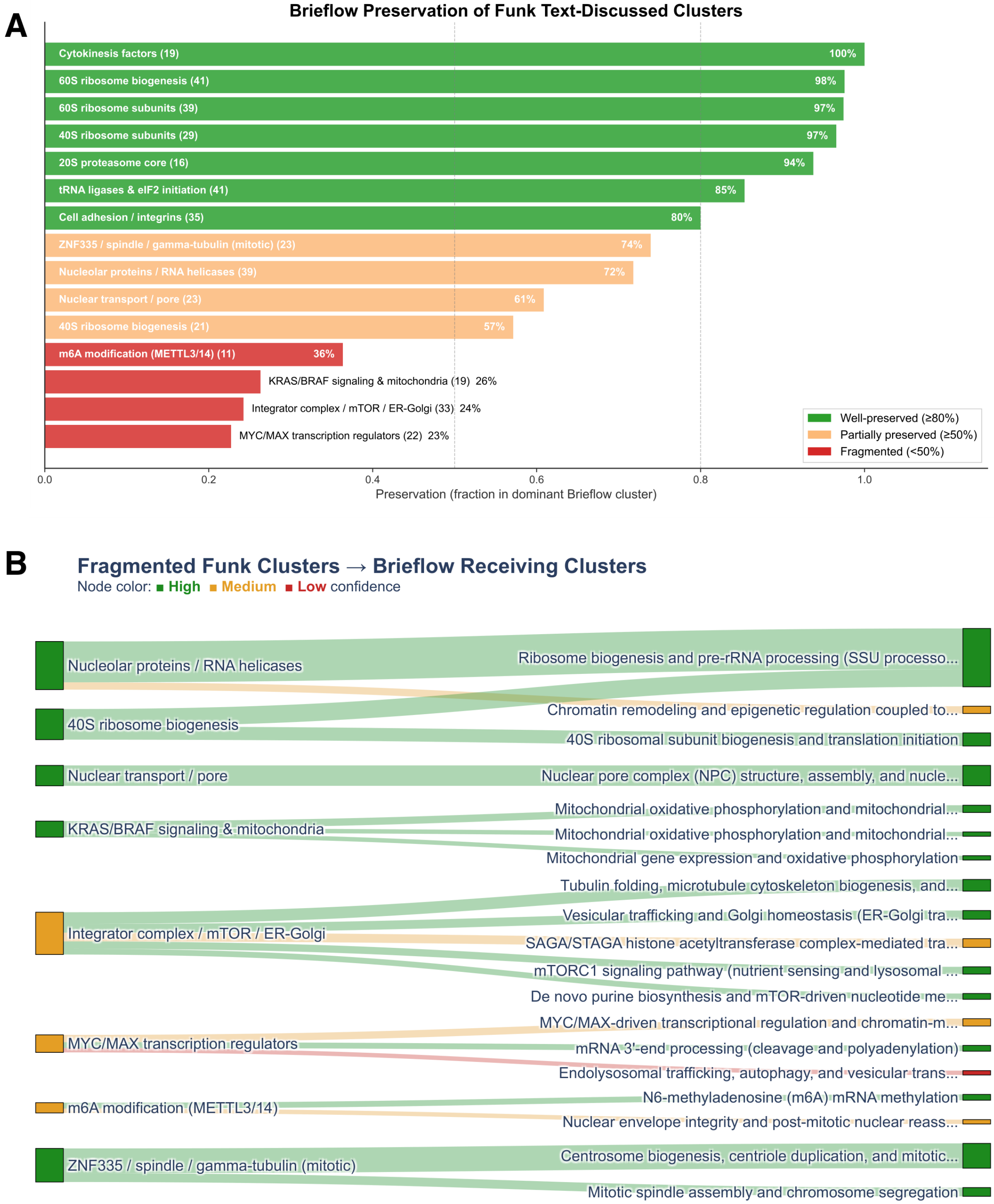

Supplementary Fig. 8: Preservation of Funk et al. biological findings in Brieflow clustering. a. Preservation analysis of the 15 Funk et al. text-discussed clusters, showing the fraction of each cluster's genes that co-occur in a single dominant Brieflow cluster. Clusters are colored by preservation level: well-preserved (≥80%, green), partially preserved (≥50%, yellow), or fragmented (<50%, red). b.

Redistribution of genes from fragmented Funk et al. clusters into Brieflow clusters. Left nodes represent original Funk et al. clusters; right nodes represent receiving Brieflow clusters, colored by MozzareLLM pathway confidence (high, medium, or low). The majority of redistributed genes are reassigned to high-confidence Brieflow modules.

Supplementary Fig. 9

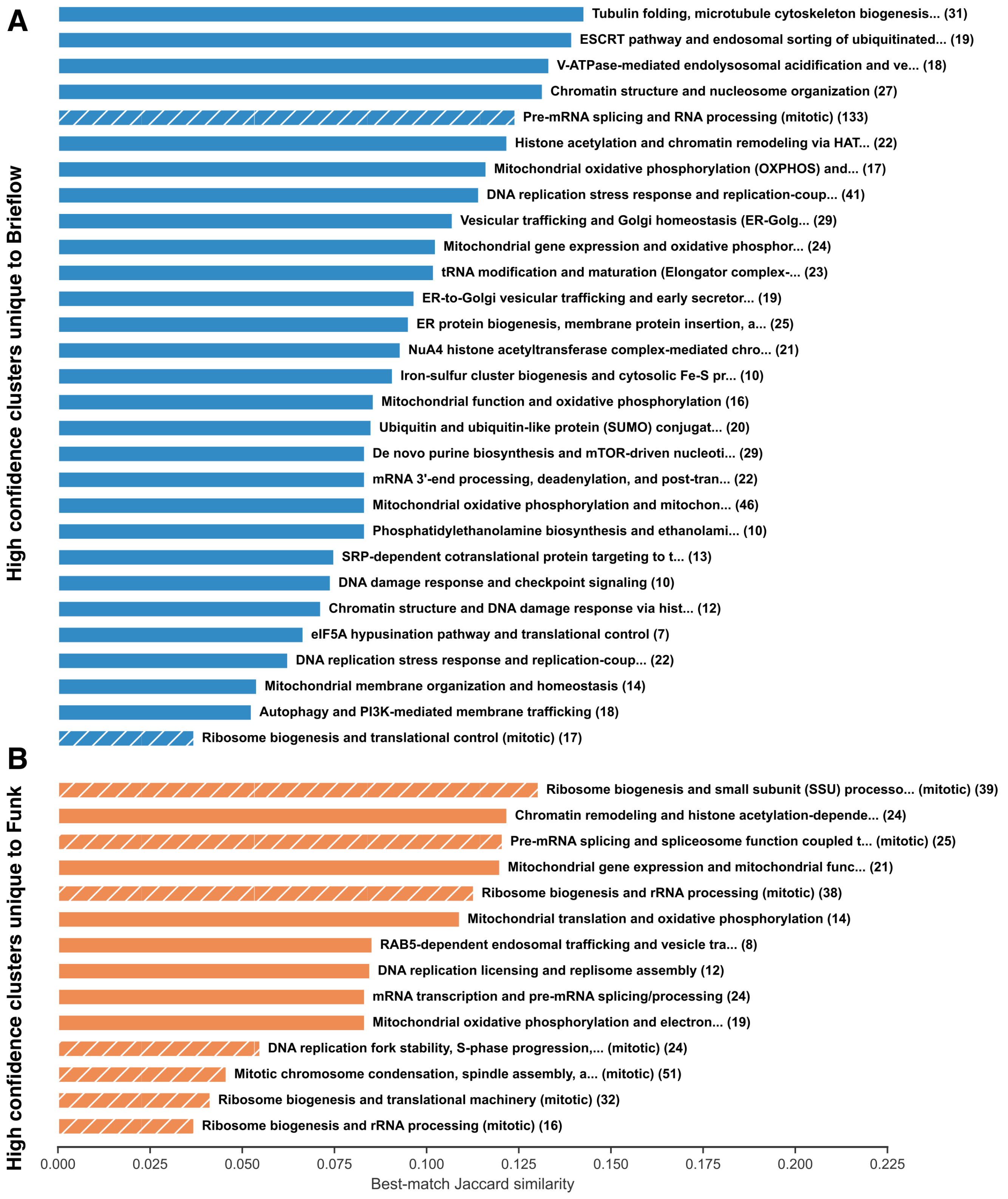

Supplementary Fig. 9: Novel functional programs resolved by Brieflow. a. High-confidence Brieflow clusters with no adequate match (Jaccard < 0.15) in the Funk et al. clustering, ranked by best-match Jaccard similarity. Each bar is annotated with the cluster pathway name, size, and lists of novel-role and uncharacterized genes. b. High-confidence Funk et al. clusters with no adequate

match in the Brieflow clustering, shown in the same format. The asymmetry (29 Brieflow-unique vs. 14 Funk-unique clusters) reflects Brieflow's finer functional resolution.
