# Supplementary material for: Brieflow: an integrated computational pipeline for high-throughput analysis of optical pooled screening data
Source: Nat Commun. 2026 May 30;17:6997. doi: 10.1038/s41467-026-73643-7 (PMC13392126; doi:10.1038/s41467-026-73643-7)
Supplement: Supplementary file 2 — Description of Additional Supplementary Files [file 41467_2026_73643_MOESM2_ESM.pdf]

Description of Additional Supplementary Files

File Name: Supplementary Data 1
Description: Brieflow MozzareLLM cluster annotations: cluster-level pathway assignments and gene-level classifications (established, novel-role, uncharacterized) with prioritization scores for interphase and mitotic populations

File Name: Supplementary Data 2
Description: Funk et al. MozzareLLM cluster annotations: same structure as Supplementary Data 1, applied to Funk et al. clustering results

File Name: Supplementary Data 3
Description: MitoCarta3.0 validation of mitochondrial sub-modules identified by Brieflow and Funk et al., including MitoCarta membership and pathway annotations for each gene

File Name: Supplementary Data 4
Description: Cluster preservation analysis tracking how 54 Funk et al. clusters map to Brieflow clusters, including preservation scores and dominant cluster assignments

File Name: Supplementary Data 5
Description: Bidirectional Jaccard similarity indices between all high-confidence Brieflow and Funk et al. clusters for interphase and mitotic populations
