# Supplementary material for: Brieflow: an integrated computational pipeline for high-throughput analysis of optical pooled screening data
Source: Nat Commun. 2026 May 30;17:6997. doi: 10.1038/s41467-026-73643-7 (PMC13392126; doi:10.1038/s41467-026-73643-7)
Supplement: Supplementary file 8 — Reporting Summary [file 41467_2026_73643_MOESM8_ESM.pdf]

Corresponding author(s): Iain M. Cheeseman

Last updated by author(s): Aprl 10, 2026

Reporting Summary

Nature Portfolio wishes to improve the reproducibility of the work that we publish. This form provides structure for consistency and transparency in reporting. For further information on Nature Portfolio policies, see our Editorial Policies and the Editorial Policy Checklist.

Statistics

For all statistical analyses, confirm that the following items are present in the figure legend, table legend, main text, or Methods section.

n/a	Confirmed	
☐	☒	The exact sample size (*n*) for each experimental group/condition, given as a discrete number and unit of measurement
☐	☒	A statement on whether measurements were taken from distinct samples or whether the same sample was measured repeatedly
☐	☒	The statistical test(s) used AND whether they are one- or two-sided<br>*Only common tests should be described solely by name; describe more complex techniques in the Methods section.*
☒	☐	A description of all covariates tested
☒	☐	A description of any assumptions or corrections, such as tests of normality and adjustment for multiple comparisons
☐	☒	A full description of the statistical parameters including central tendency (e.g. means) or other basic estimates (e.g. regression coefficient) AND variation (e.g. standard deviation) or associated estimates of uncertainty (e.g. confidence intervals)
☐	☒	For null hypothesis testing, the test statistic (e.g. *F*, *t*, *r*) with confidence intervals, effect sizes, degrees of freedom and *P* value noted<br>*Give P values as exact values whenever suitable.*
☒	☐	For Bayesian analysis, information on the choice of priors and Markov chain Monte Carlo settings
☒	☐	For hierarchical and complex designs, identification of the appropriate level for tests and full reporting of outcomes
☐	☒	Estimates of effect sizes (e.g. Cohen's *d*, Pearson's *r*), indicating how they were calculated

Our web collection on statistics for biologists contains articles on many of the points above.

Software and code

Policy information about availability of computer code

Data collection	All data was acquired using NIS-Elements AR using a Nikon Ti-2 inverted epifluorescence microscope with automated stage control, hardware autofocus, and an Iris 9 sCMOS camera.
Data analysis	Data was analyzed using the brieflow codebase (https://github.com/cheeseman-lab/brieflow), using a specific instance of brieflow-analysis (https://github.com/cheeseman-lab/brieflow-analysis), found here: https://github.com/cheeseman-lab/aconcagua-analysis.

For manuscripts utilizing custom algorithms or software that are central to the research but not yet described in published literature, software must be made available to editors and reviewers. We strongly encourage code deposition in a community repository (e.g. GitHub). See the Nature Portfolio guidelines for submitting code & software for further information.

Data

Policy information about availability of data

All manuscripts must include a data availability statement. This statement should provide the following information, where applicable:

- Accession codes, unique identifiers, or web links for publicly available datasets
- A description of any restrictions on data availability
- For clinical datasets or third party data, please ensure that the statement adheres to our policy

The raw imaging data in TIFF format used in this study were originally generated by Funk et al. and are deposited in BioImage Archive under accession S-BIAD394

(https://www.ebi.ac.uk/biostudies/BioImages/studies/S-BIAD394). The raw .nd2 files are deposited in BioImage Archive. Processed results including cluster groupings and feature matrices are publicly accessible at https://screens.wi.mit.edu/aconcagua. Source data are provided with this paper.

Research involving human participants, their data, or biological material

Policy information about studies with human participants or human data. See also policy information about sex, gender (identity/presentation), and sexual orientation and race, ethnicity and racism.

Reporting on sex and gender	N/A
Reporting on race, ethnicity, or other socially relevant groupings	N/A
Population characteristics	N/A
Recruitment	N/A
Ethics oversight	N/A

Note that full information on the approval of the study protocol must also be provided in the manuscript.

Field-specific reporting

Please select the one below that is the best fit for your research. If you are not sure, read the appropriate sections before making your selection.

☒ Life sciences ☐ Behavioural & social sciences ☐ Ecological, evolutionary & environmental sciences

For a reference copy of the document with all sections, see nature.com/documents/nr-reporting-summary-flat.pdf

Life sciences study design

All studies must disclose on these points even when the disclosure is negative.

Sample size	No statistical method was used to predetermine sample size. The dataset comprises all available cells from the previously published Vesuvius screen (Funk et al. 2022).
Data exclusions	No data were excluded from the analyses beyond the quality-control filtering steps described in the Aggregate module (outlier removal via Local Outlier Factor with contamination = 0.01; missing-value filtering at thresholds of 0.05 for columns and 0.01 for rows).
Replication	For benchmarking comparisons (spot detection, segmentation, feature extraction), one tile was randomly selected per plate (n = 8 tiles total) to provide representative sampling across experimental batches. Results were consistent across tiles.
Randomization	The experiments were not randomized. This study involves computational reanalysis of a previously published dataset.
Blinding	The investigators were not blinded to allocation during experiments and outcome assessment, as this study involves computational reanalysis of previously published data where blinding is not applicable.

Reporting for specific materials, systems and methods

We require information from authors about some types of materials, experimental systems and methods used in many studies. Here, indicate whether each material, system or method listed is relevant to your study. If you are not sure if a list item applies to your research, read the appropriate section before selecting a response.

Materials & experimental systems

n/a	Involved in the study
☒	☐ Antibodies
☒	☐ Eukaryotic cell lines
☒	☐ Palaeontology and archaeology
☒	☐ Animals and other organisms
☒	☐ Clinical data
☒	☐ Dual use research of concern
☒	☐ Plants

Methods

n/a	Involved in the study
☒	☐ ChIP-seq
☒	☐ Flow cytometry
☒	☐ MRI-based neuroimaging

Plants

Seed stocks	N/A
Novel plant genotypes	N/A
Authentication	N/A
