# Supplementary material for: Brieflow: an integrated computational pipeline for high-throughput analysis of optical pooled screening data
Source: Nat Commun. 2026 May 30;17:6997. doi: 10.1038/s41467-026-73643-7 (PMC13392126; doi:10.1038/s41467-026-73643-7)
Supplement: Supplementary file 9 — Transparent Peer Review file [file 41467_2026_73643_MOESM9_ESM.pdf]

nature portfolio

Peer Review File

Brieflow: An Integrated Computational Pipeline for High-Throughput Analysis of Optical Pooled Screening Data

Corresponding Author: Dr Iain M. Cheeseman

This file contains all reviewer reports in order by version, followed by all author rebuttals in order by version.

Version 0:

Reviewer comments:

Reviewer #2

(Remarks to the Author)
Here, Di Bernardo et al. present Brieflow, a computational pipeline for integrated analysis of Optical Pooled Screening (OPS) image data. Brieflow is built around a modular framework that spans all steps of OPS analysis: image preprocessing, barcode decoding, phenotypic feature extraction, and cluster analysis. The key conceptual advance lies in the unification of these previously fragmented steps into a coherent, end-to-end pipeline. By automating complex OPS analyses, Brieflow lowers the barrier to entry for non-experts, while its modular architecture allows expert users to adapt or reuse individual components for customized applications.

In addition, the authors introduce MozzareLLM, an AI tool that annotates gene clusters with biological ontology terms and prioritizes candidate genes for follow-up experiments. This represents a novel and relevant approach to streamlining hypothesis generation from high-dimensional imaging data.

Overall, the work is both novel and timely. The OPS technology is still emerging but has a broad potential across cell biology. However, the current analytical ecosystem for OPS remains sparse. Computational frameworks like Brieflow are critically needed — not only to improve accessibility, but also to enable data harmonization and reproducibility, much like the role early software tools played in catalyzing the widespread adoption of single-cell RNASeq. The authors provide a well-documented GitHub repository, alongside a user-friendly interactive visualization dashboard. MozzareLLM, in its own right, is a compelling tool for accelerating biological insight from unsupervised clustering.

This study presents a solid computational foundation and will be of high interest to the readers of Nature Communications. Brieflow is definitely worth a place on the cheese board. However, some improvements are needed to strengthen the manuscript’s quantitative claims and to better discuss the generalizability of Brieflow beyond the authors' dataset. I hope the following comments will be helpful in that regard.

MAJOR COMMENTS

1. Strengthening the author’s claims with quantitative analyses.

Throughout the text, the authors describe their pipeline as “precise”, “robust” or “accurate”, but quantitative evidence supporting these claims is limited. While the figures provide helpful illustrations, more rigorous quantitative benchmarking is needed to substantiate the pipeline’s performance. Specifically:

1.1) Sequencing-by-synthesis: Could the authors provide estimates of precision and recall for the barcode decoding step? This could be accomplished using cells transduced with a known gRNA, or with synthetic in situ sequencing datasets where the ground truth is available.
1.2) Phenotyping: how is the “precise alignment of fluorescence channels” (l. 179) conducted? How is precision measured?
1.3) Phenotyping / segmentation accuracy: it is great the authors compare different segmentation algorithms, and the images in Figure 2E give a general idea of comparative performance. But the statement that "Cellpose exhibited superior segmentation accuracy" (l.189) requires quantitative support. This could be addressed by benchmarking the 8 image tiles against a human expert–annotated ground truth, and reporting % nuclei properly segmented and % cell membrane properly

segmented. The current metric (% of cells containing at least one nucleus) is helpful, but not sufficient for evaluating segmentation fidelity.
1.4) Phenotyping / marker generalization: The segmentation results are demonstrated using Actin and DAPI, which are relatively robust markers. However, Brieflow is intended to generalize across markers. Can the authors provide segmentation performance for additional channels (e.g., Tubulin, H2AX) to validate broader applicability?
1.5) Feature extraction: it makes sense for the authors to use a Python-based CellProfiler emulator. I appreciated that the authors report comparison data with vanilla Cellprofiler features. However, the discrepancies in Extended Data Fig. 4G-H raise questions. Can the authors elaborate on the causes of this divergence? It would be helpful to clarify whether this reflects known limitations in the emulator's functionality (as suggested in the Methods), or differences in implementation logic (e.g., how features are computed). More context here will help readers decide when the emulator is appropriate for their use cases.
1.6) Merge: can registration performance be quantified? This could be done from synthetic data, or by comparing to “human-curated ground truth” with annotated image pairs. Without more context, it is difficult for the reader to know what to make of the reported 32% loss of cells at the registration step.
1.7) Aggregation: it is not clear in the main text how aggregation is performed (mean, median etc). This is explained in the Methods but deserves being explicit in the main text to provide context for non-experts.
1.8) Overall, it would be useful to provide a head-to-head quantitative comparison of the new analysis derived from Brieflow with the author’s original analysis in Funk et al 2022. I appreciated the estimation of Precision-Recall (PR) in Fig 4F. I wonder if the same analysis could be done from the original clusters in Funk et al. Presumably, the area under the PR curve would be lower in Funk et al?

2. Generalizability of Brieflow beyond the authors’ dataset

The reanalysis of the “Vesuvius” HeLa dataset clearly demonstrates Brieflow’s capabilities. However, since the pipeline is positioned as a general-purpose OPS analysis framework, further evidence is needed to support its performance across diverse datasets and experimental designs.

2.1) At the very least, the applicability to Zombie/Perturbview in-situ sequencing data should be characterized. The field is moving to PerturbView ISS, and this should be supported. Have the authors tested Brieflow on any Zombie/PerturbView datasets? Even a brief validation (e.g., successful barcode identification) would strengthen confidence in generalizability.

2.2) Application to an independent dataset: ideally, Brieflow should be demonstrated on an additional OPS dataset, preferably generated by an external group. If raw data access is limiting, the authors could instead show results on a different in-house dataset (beyond the HeLa Vesuvius screen) to illustrate performance across cell types, markers, or library complexity. The analysis does not need to be exhaustive.

3. Limitation of the study.

It would be great to add a “limitation of this study” section to the discussion. What could be improved? What is still missing? What next functionalities could be added? This would provide useful context to the reader without taking anything away from the author’s contribution.

4. Github repo & Streamlit app.

4.1) The Github repo is clear and well annotated. However, I did not notice any demo data to quickly run the pipeline without the user having their own dataset (sorry if I missed it). If that is the case, it would be great to include one. Consider also adding a high-level summary at the top of the repo’s readme to introduce what OPS is, and what the package is used for. This would help coders who might come across the repo understand better what the tool is for.
4.2) The visualization app is very useful, but basic info about how to use the app’s functionalities is required. You can add this to the readthedocs, and provide a link in the Streamlit app.
4.3) Double-check all functionalities of the app. At least when I tried it, MozareLLM seemed to only work when the “interphase” cell class is selected (i.e. not with “all” or “mitotic”).

MINOR COMMENTS (feel free to ignore):

1. MozareLLM is a cool tool that could be useful beyond Brieflow– mention it in the abstract so it can be more easily picked up in searches?
2. I might have missed it, but is Ext. Figure 1 cited in the text? Maybe cite it in the first paragraph?
3. L. 45: “simplistic” sounds deprecating – fitness-based screens can provide very useful insights. Use “simple” instead?
4. Fig. 1 might not be the most intuitive for non-experts. Consider adding more explicit visual details about the different steps of OPS (cf. Figure 1 in Funk et al?).
5. L. 106: for non-experts, can you provide more intuitive context regarding the advantages of Snakefile, and why they are useful for Brieflow?
6. L. 158: "standard method based on statistical signal detection" - this requires more info, or at least a reference?
7. The panel arrangement in Fig. 3 is a bit confusing. Consider re-organizing.
8. The “aggregation” aspect is not intuitively captured in Figure 3C. Consider adding two colored columns to the “Cells” matrix at the top: “Barcode” and “Genes”, with multiple cells sharing the same gene. Then the “Perturbation” matrix at the bottom could be smaller and only show the “aggregated” genes with the same colors.

9. L. 212 what do you mean by “cellular objects”? How does the number 69,065,844 compare to the “70+ million cells identified” mentioned l. 236?
10. L. 238: the “triangulation hashing” approach is not necessarily intuitive for non-experts. Consider adding give a more intuitive explanation re: what triangular hashing does. Which channels does it use?
11. Ll. 395-396: I got confused by the switch of naming nomenclature between C18orf21/NEPRO and RMP24/RMP64. Keep C18orf21/NEPRO nomenclature throughout to be consistent?
12. Ext. Data Figure 9: why are there only 4 genes mentioned in cluster 60 in panel A, when more genes from that cluster are presented in panel B? Probably because panel A only covers only vesicular trafficking genes? The legend for panel A is a bit confusing, mentioning “The refinement reveals distinct vesicular transport modules including separated exocyst complex and CCR4-NOT components” which is not shown in that Figure.
13. It is great that Brieflow starts filling the holes in the Swiss cheese ecosystem of OPS data analysis.

(Remarks on code availability)
(Comments included in the main review)

Reviewer #3

(Remarks to the Author)
This manuscript introduces Brieflow, an open-source, end-to-end pipeline for analyzing fixed-cell OPS data. The workflow spans raw image conversion coupled with illumination correction, in-situ barcode decoding, phenotypic feature extraction, genotype–phenotype merging, aggregation and clustering for downstream interpretation and visualization. Using a previously published HeLa OPS dataset as a case study, the authors report improved recovery of pathway-level structure and provide an LLM-assisted interpretation component.

Computational analysis of OPS has been a nontrivial barrier to entry for many labs. Overall, the package is thoughtfully engineered, the documentation is clear, and the notebooks are helpful for onboarding. While some functionality is limited in scope, the work has practical value for groups adopting OPS. The paper is acceptable with some minor revisions.

1. The manuscript would benefit from an explicit statement of data-collection assumptions. For example, many instrument setups do not guarantee one-to-one tile correspondence across cycles; thus, the current solution may not suit all microscope configurations. A related issue is compatibility with the multiple-round phenotyping via iterative staining. Such limitations should be clearly stated to communicate the software's scope.
2. The reanalysis of HeLa data should be evaluated and visualized more thoroughly. Embedding and clustering are sensitive to method choice and hyperparameters. For example, if Leiden clustering is applied to PHATE embeddings, varying the embedding dimensionality and other PHATE parameters will yield different clustering results (in particular, if clustering were performed on two-dimensional embeddings, substantial information would be discarded). While the Methods section describes evaluation against knowledgebases, please also assess embedding quality and present these results in the main figures.
3. Similarly, many claims about improvement over the previous pipelines are not rigorously compared. While the current pipeline provides helpful case studies, it is also possible that the prior Vesuvius dataset captures other biological processes more effectively. A systematic, dataset-wide comparison demonstrating that embedding and clustering results are generally comparable or improved would help, before emphasizing improvements in specific clusters. If reanalyzing the Vesuvius dataset is not feasible, please temper and clearly qualify these claims.
4. To support these points, consider merging the current Fig. 2 and Fig. 3 into a single figure, and dedicate one figure exclusively to QC/benchmarking on the reanalysis.
5. Line 348 “a mean of 1.3 completely uncharacterized genes per cluster” reflects library composition rather than a property of MozzareLLM. Please adjust the wording of this section to avoid implying a discovery specific to MozzareLLM.

Minor points
- line 345: When cluster labels are permuted, how many “High confidence” biological processes would be detected? I suggest adding such a baseline.
- While the manuscript emphasizes modularity, there appears to be a learning curve for adding new functions to modules. I recommend organizing the repository and adding brief instructions wherever possible.
- Small test images downloadable from GitHub are very helpful for new users. Consider referencing them explicitly in the Data Availability section.
- Extended Data Fig. 2 currently lacks illumination-correction results.

(Remarks on code availability)
The documentation was clear for installation and running the test that they prepared. I did not test it for real data.

Version 1:

Reviewer comments:

Reviewer #2

(Remarks to the Author)
Congratulations to the authors for the revised version of this manuscript. My comments have been successfully addressed

during revisions, and I fully support publication.

I have a couple of final clarification questions. These will not require any further review, but I would encourage the authors to address them as they prepare the final version of their manuscript:
- Review all Figure legends and maximize explicit explanations. It took me a while to understand some figure panels. Adding more info in the legend would be quite helpful. In some instances, the definition of some terms might not be intuitive to a general-audience reader. For example "mapped cells" in Figure 2C.
- Fig. 3B "Representative snapshot of merge approach" graph - it took me a while to understand what you are trying to show in that panel. Consider extending the legend.
- Fig. 3D: how were the specific principal components chosen? The figure seems to imply a correspondence between PCs and the original features such as mean DAPI intensity. If so, could you make this explicit in the legend and explain how it was determined? If not, and PCs were merely chosen to show normalization, consider saying that.
- l. 428 "MozzareLLM identified distinct high-confidence biological processes" -> how is "high confidence" defined?
- I had a hard time understanding what is shown in Fig. 5C and D. Be explicit about what each triangle of the heatmap represents, and label the axes? Is this showing the difference between PCA at the single-cell level vs after gene-level averaging? The corresponding part of the text could be clarified accordingly. The concepts or "raw interpretable features" (l. 497) or "PCA-based embeddings (l. 500) are not very intuitive to understand.
- Ensure that font size in all Figure panels is above a threshold. Some text is very small (e.g. axes labels).

(Remarks on code availability)
I did inspect the code and found it well-documented and clearly organized. The Readthedocs documentation is helpful. I did not run the code on real data.

Reviewer #3

(Remarks to the Author)
The authors have addressed my comments. I support publication in Nature Communications.

(Remarks on code availability)
I have installed and successfully ran their tests but have not applied to other datasets.

Open Access This Peer Review File is licensed under a Creative Commons Attribution 4.0 International License, which permits use, sharing, adaptation, distribution and reproduction in any medium or format, as long as you give appropriate credit to the original author(s) and the source, provide a link to the Creative Commons license, and indicate if changes were made.
In cases where reviewers are anonymous, credit should be given to 'Anonymous Referee' and the source.
The images or other third party material in this Peer Review File are included in the article's Creative Commons license, unless indicated otherwise in a credit line to the material. If material is not included in the article's Creative Commons license and your intended use is not permitted by statutory regulation or exceeds the permitted use, you will need to obtain permission directly from the copyright holder.
To view a copy of this license, visit https://creativecommons.org/licenses/by/4.0/

We thank the reviewers for their thoughtful and constructive feedback on our manuscript. We are pleased that the reviewers recognize the value of Brieflow as a comprehensive computational framework for optical pooled screening analysis. In response to the reviewers' suggestions, we have made substantial improvements to the manuscript including additional analyses, enhanced documentation, and clarification of key methodological details.

Since the initial submission, Brieflow has been successfully deployed across more than 10 distinct optical pooled screening datasets generated in multiple laboratories. To support the growing user community, we have established a Discord server that facilitates technical support and knowledge sharing among researchers implementing Brieflow on diverse experimental systems. This real-world deployment has provided valuable validation of the pipeline's robustness and has driven continued development of new capabilities.

In response to reviewer feedback, we have implemented several major enhancements to Brieflow's functionality:

- *Extended barcode detection capabilities*. We now provide support for recombination-detected barcodes in addition to traditional sequencing-by-synthesis approaches.
- *Broader microscopy platform compatibility*. We have incorporated native support for TIFF-based formats from Phenix and Squid microscopy systems, expanding beyond ND2 files.
- *Multiple registration algorithms*. We have now implemented alternative methods for aligning data between imaging modalities, providing optimized approaches for different experimental configurations.
- *Cell state classification*. We have created a new “Classify” module for training custom machine learning classifiers to partition cells into relevant subpopulations (e.g., mitotic vs. interphase), enabling cell-state-specific downstream analyses.
- *Statistical validation frameworks*. We have incorporated bootstrapping approaches to rigorously test feature significance relative to non-targeting controls.
- *Single-cell perturbation scoring*. We have incorporated methods to distinguish individual cell deviations from control distributions, enabling single-cell resolution analyses.

These enhancements, combined with the improvements and additions detailed below, substantially strengthen Brieflow's applicability across diverse experimental contexts and biological questions.

Below, we provide detailed point-by-point responses to each reviewer comment.

Reviewer #2

Brieflow is definitely worth a place on the cheese board.

We appreciate this incredibly cheesy comment.

Strengthening the author's claims with quantitative analyses. Throughout the text, the authors describe their pipeline as "precise", "robust" or "accurate", but quantitative evidence supporting these claims is limited. While the figures provide helpful illustrations, more rigorous quantitative benchmarking is needed to substantiate the pipeline's performance.

We thank the reviewer for this suggestion. We have toned down unsubstantiated adjectives throughout the manuscript and replaced them with specific quantitative metrics wherever possible. We have also substantially expanded the Methods section with detailed algorithmic descriptions, parameter choices, and performance benchmarks for each module. We address each specific sub-point below.

1. Sequencing-by-synthesis: Could the authors provide estimates of precision and recall for the barcode decoding step? This could be accomplished using cells transduced with a known gRNA, or with synthetic in situ sequencing datasets where the ground truth is available.

The original manuscript already included a head-to-head comparison of the standard spot detection method versus Spotiflow. We have expanded this benchmarking with additional detail. In addition, to directly address the question of ground-truth validation, we have applied Brieflow's SBS module to an independent in situ sequencing (ISS) dataset containing three known barcode constructs sequenced via T7-based ISS chemistry across three cycles. We have added these new analyses to the results section:

Expanded SBS section: "In benchmarking analysis across eight randomly selected tiles (one per plate), the standard method detected substantially more initial peaks than Spotiflow (48,133 vs. 13,344 peaks on average per tile). However, after quality filtering, the standard method yielded 9,486 high-quality reads per tile compared to 13,344 for Spotiflow (Fig. 2c). Critically, read mapping fractions were higher with the standard method (71.8% vs. 57.6%), although the proportion of cells successfully assigned to a single gene target remained comparable between approaches (66.5% vs. 66.9%). Processing with Spotiflow required substantially more time, averaging 40.3 seconds per tile compared to just 0.87 seconds with the standard method (Extended Data Fig. 3a)."

New validation paragraph: "To validate Brieflow's generalizability, we applied the SBS module to an independent in situ sequencing dataset with three known barcode constructs sequenced across three cycles using T7-based ISS chemistry. Brieflow accurately mapped 85.0% of cells to the three known constructs, with 78.2% uniquely assigned to a single construct (Extended Data Fig. 3d)."

2. Phenotyping: how is the "precise alignment of fluorescence channels" (l. 179) conducted? How is precision measured?

We have replaced "precise" with a detailed description of the alignment methodology and its evaluation. This section now reads:

"Channel alignment was performed using phase cross-correlation for sub-pixel precision, with integrated notebook visualizations enabling manual inspection of registration accuracy. For high-magnification or multi-round imaging contexts where automated alignment may be insufficient, the module supports a two-stage approach combining user-specified coarse offsets with automated sub-pixel refinement. Custom channel-specific offsets can additionally be specified to correct for systematic chromatic aberrations."

3. Phenotyping / segmentation accuracy: it is great the authors compare different segmentation algorithms, and the images in Figure 2E give a general idea of comparative performance. But the statement that "Cellpose exhibited superior segmentation accuracy" (l.189) requires quantitative support. This could be addressed by benchmarking the 8 image tiles against a human expert-annotated ground truth, and reporting % nuclei properly segmented and % cell membrane properly segmented. The current metric (% of cells containing at least one nucleus) is helpful, but not sufficient for evaluating segmentation fidelity.

We have replaced the claim of "superior segmentation accuracy" with quantitative retention metrics from a practical comparison designed to mirror the user experience within Brieflow. Rather than benchmarking against expert-annotated ground truth — which would not reflect how researchers actually use the pipeline — we evaluated three segmentation approaches under the same conditions a Brieflow user would encounter: running each method on representative tiles and measuring downstream retention through the pipeline's reconciliation steps. This design philosophy is deliberate: Brieflow's goal is to provide and compare segmentation methods, not to benchmark them against ground truth, which users would not have available. The retention metrics — measuring the proportion of detected objects that survive downstream reconciliation of nuclear and cellular masks — provide a practically meaningful measure of segmentation quality within the pipeline context. We have additionally replaced the microSAM model with the more used Cellpose4 SAM model. This section now reads:

"In comparative benchmarking on one random imaging tile per plate (8 tiles total), Cellpose (cyto3) achieved high retention rates (93.0% nuclei, 93.7% cell retention) with a moderate processing time (186 seconds per tile). Cellpose4 (CPSAM) achieved comparable segmentation but with expectedly longer processing times (1,161 seconds per tile); however, CPSAM offers strong performance for non-traditional morphologies such as elongated RPE1 cells, making it a valuable alternative for challenging cell types. StarDist was most computationally efficient (110 seconds per tile) but achieved lower retention rates (16.7% nuclei, 35.1% cell retention), with many detected objects lost during downstream reconciliation (Fig. 2e, Extended Data Fig. 4a-d). Based on these results, we selected Cellpose (cyto3) for the Vesuvius reanalysis. Notebook visualizations enable users to test segmentation approaches on representative tiles before committing to full-scale processing, facilitating informed method selection for each experimental context."

4. Phenotyping / marker generalization: The segmentation results are demonstrated using Actin and DAPI, which are relatively robust markers. However, Brieflow is intended to

generalize across markers. Can the authors provide segmentation performance for additional channels (e.g., Tubulin, H2AX) to validate broader applicability?

We have clarified that segmentation operates on a user-selected channel, with resulting masks applied across all channels for feature extraction. Marker generalization is therefore a property of the feature extraction step, not the segmentation step.

5. Feature extraction: it makes sense for the authors to use a Python-based CellProfiler emulator. I appreciated that the authors report comparison data with vanilla Cellprofiler features. However, the discrepancies in Extended Data Fig. 4G-H raise questions. Can the authors elaborate on the causes of this divergence? It would be helpful to clarify whether this reflects known limitations in the emulator's functionality (as suggested in the Methods), or differences in implementation logic (e.g., how features are computed). More context here will help readers decide when the emulator is appropriate for their use cases.

We have now expanded a discussion of feature extraction divergences to help readers assess appropriateness for their use cases. This section now reads:

"Benchmarking against cp_measure revealed strong agreement for interpretable intensity and shape features, while features sensitive to local variations—particularly radial distribution features—showed divergence between implementations (Extended Data Fig. 4g-h). These discrepancies likely reflect differences in implementation details for complex multi-step computations rather than fundamental algorithmic differences. Furthermore, the cp_measure implementation required an average processing time of nearly 5 hours per tile, which is impractical for high-content screens."

6. Merge: can registration performance be quantified? This could be done from synthetic data, or by comparing to "human-curated ground truth" with annotated image pairs. Without more context, it is difficult for the reader to know what to make of the reported 32% loss of cells at the registration step.

We have substantially expanded the “Merge” section to provide quantitative context for cell loss, including validation that loss is concentrated at tile boundaries (where duplicate detections are expected) rather than reflecting registration failures. This section now states:

"The approximately 25% cell loss during merge primarily reflects intentional filtering of cells at tile boundaries, where overlapping fields of view result in duplicate cell detections. This deduplication step is essential for preventing the same physical cell from contributing multiple observations to downstream analyses, and the retention rates are consistent with the expected overlap between adjacent tiles in the imaging configuration. For a representative area, the triangle hash approach achieves a 97% match rate in non-overlapping tile regions compared to 53% in overlapping regions, directly confirming that cell loss is concentrated at tile boundaries where duplicate detections are expected rather than reflecting registration failures (Extended Data Fig. 5b-c)."

We have also implemented an alternative well-level stitching approach for screens where cell density is too low for robust tile-by-tile registration: "For experimental conditions where cell density is low, the module implements an alternative well-level

stitching approach that first assembles tiles into complete well mosaics using recorded stage coordinates, then applies the same triangulation-based matching strategy to the complete cell population within each well."

7. Aggregation: it is not clear in the main text how aggregation is performed (mean, median etc). This is explained in the Methods but deserves being explicit in the main text to provide context for non-experts.

We have now added additional details to this section of the text. "For the final aggregation step, cellular measurements are collapsed to the perturbation level using median aggregation, which provides robustness to outliers common in single-cell data; alternative methods including mean and trimmed mean are also supported."

We have also added a description of the single-cell PCA design choice and TVN batch correction to the main text, with quantitative validation: "TVN batch correction was validated by ANOVA F-test for batch-associated variation, with p-values increasing from highly significant levels (10^{-3} for interphase) to non-significant levels (0.52 for interphase), confirming successful removal of technical variation (Fig. 3d)."

8. Overall, it would be useful to provide a head-to-head quantitative comparison of the new analysis derived from Brieflow with the author's original analysis in Funk et al 2022. I appreciated the estimation of Precision-Recall (PR) in Fig 4F. I wonder if the same analysis could be done from the original clusters in Funk et al. Presumably, the area under the PR curve would be lower in Funk et al?

We thank the reviewer for this suggestion, which prompted the most substantial additions to the manuscript. We now provide a comprehensive, systematic head-to-head comparison with the analysis described in Funk et al. 2022 across multiple dimensions.

First, we have added benchmarking data relative to standardized definitions of protein grouping. This text now states: "Compared with the Funk et al. clustering at their optimal resolutions (k=10 interphase, k=9 mitotic; both producing 222 clusters), Brieflow's interphase clustering showed a higher STRING F1 score (0.098 vs. 0.067), CORUM enrichment (33.0% vs. 23.9%), and KEGG enrichment (18.1% vs. 13.5%). Mitotic clustering showed higher STRING F1 (0.065 vs. 0.044) but lower CORUM (5.4% vs. 7.2%) and KEGG enrichment (3.2% vs. 5.4%)."

We have also used our MozzareLLM strategy to compare these datasets: "For interphase clusters (n=227 at k=12), MozzareLLM identified distinct high-confidence biological processes for 73 clusters (32.2%), compared with 52 high-confidence clusters (23.4% of 222 clusters) from the original Funk et al. analysis at its optimal resolution. These 73 high-confidence clusters captured 1,874 genes compared with Funk et al.'s 1,114 genes, a 68% increase in genes assigned to interpretable biological pathways."

Finally, we have added a new Results section titled 'Reanalysis with Brieflow recovers mitochondrial clusters' that leads with the mitochondrial biology finding (five Brieflow clusters vs. four Funk clusters) and includes systematic cross-pipeline preservation analysis (50/57 curated clusters annotated, 77.2% partially preserved) and Jaccard-based divergence analysis (35% of Brieflow clusters unique vs. 21% of Funk clusters), with supporting data in Supplemental Tables S3–S8.

Generalizability of Brieflow beyond the authors' dataset. The reanalysis of the "Vesuvius" HeLa dataset clearly demonstrates Brieflow's capabilities. However, since the pipeline is positioned as a general-purpose OPS analysis framework, further evidence is needed to support its performance across diverse datasets and experimental designs.

9. At the very least, the applicability to Zombie/Perturbview in-situ sequencing data should be characterized. The field is moving to PerturbView ISS, and this should be supported. Have the authors tested Brieflow on any Zombie/PerturbView datasets? Even a brief validation (e.g., successful barcode identification) would strengthen confidence in generalizability.

We have addressed this comment in two ways: 1) Adding PerturbView/Zombie barcode support, and 2) validating this approach on an independent in situ sequencing dataset. In particular, the results section now includes a new analysis that states:

"To validate Brieflow's generalizability, we applied the SBS module to an independent dataset generated using three known barcode constructs sequenced across three cycles using T7-based chemistry. Brieflow accurately mapped 78.2% of the segmented cells uniquely to a single construct (Extended Data Fig. 3d). PerturbView-style in situ sequencing produces single high-intensity nuclear peaks rather than distributed cytoplasmic signal; both characteristics are accommodated through Brieflow's configurable parameters for peak detection prioritization and compartment-specific read assignment."

10. Application to an independent dataset: ideally, Brieflow should be demonstrated on an additional OPS dataset, preferably generated by an external group. If raw data access is limiting, the authors could instead show results on a different in-house dataset (beyond the HeLa Vesuvius screen) to illustrate performance across cell types, markers, or library complexity. The analysis does not need to be exhaustive.

Since the initial submission, Brieflow has been deployed on more than 10 distinct OPS datasets generated across multiple laboratories, covering diverse cell types, perturbation libraries, and imaging configurations. Although a full independent dataset reanalysis was not feasible within the scope of this revision, the *in situ* sequencing validation on T7-based chemistry (described above) demonstrates generalizability of the SBS module to a different sequencing approach. We have added a “limitations” paragraph to the Discussion acknowledging that our comprehensive benchmarking focuses on a single large-scale dataset, while noting the real-world validation through multi-laboratory deployment.

11. It would be great to add a "limitation of this study" section to the discussion. What could be improved? What is still missing? What next functionalities could be added? This would provide useful context to the reader without taking anything away from the author's contribution.

We have now added a limitations section to the Discussion covering four areas. This section states that:

"The current implementation assumes one-to-one tile correspondence within phenotyping rounds and sequencing-by-synthesis cycles, a constraint satisfied by most standard microscope configurations... Registration quality depends on the ability to identify corresponding cellular landmarks across imaging rounds. The default tile-

by-tile registration performs robustly under standard screening conditions, but high-magnification screens with sparse cellular populations may not provide sufficient landmarks per tile for robust geometric matching... Our CellProfiler-inspired feature extraction, while effective for morphological profiling, reflects a broader challenge in the field: reliable methods to extract single-cell image features in a fully Pythonic format are not widely available... A central priority is aligning Brieflow with next-generation file formats (NGFF), particularly OME-Zarr, to improve interoperability with the broader imaging ecosystem."

12. 4.1) The Github repo is clear and well annotated. However, I did not notice any demo data to quickly run the pipeline without the user having their own dataset (sorry if I missed it). If that is the case, it would be great to include one. Consider also adding a high-level summary at the top of the repo's readme to introduce what OPS is, and what the package is used for. This would help coders who might come across the repo understand better what the tool is for.

We have now improved the repository with a high-level summary at the top of the README introducing OPS and Brieflow's purpose. In addition, we have made the small test image dataset that was already included in the repository easier to find. Users that would like to validate installation, test functionality, and add new features can easily use one script to download test data, and another to run Brieflow on this test data.

13. The visualization app is very useful, but basic info about how to use the app's functionalities is required. You can add this to the readthedocs, and provide a link in the Streamlit app.

We have now added documentation for the Streamlit visualization app to the readthedocs site, including descriptions of each display panel and instructions for interpreting MozzareLLM outputs. We have furthermore strongly enriched the data annotation section to cover many more details of the screen, to serve as a backbone for future data collection and annotation efforts for users of Brieflow.

14. Double-check all functionalities of the app. At least when I tried it, MozareLLM seemed to only work when the "interphase" cell class is selected (i.e. not with "all" or "mitotic").

This has been resolved. The app now clearly indicates which MozzareLLM annotations are available for each cell class and resolution combination, so users are not confused in searching for these. The revised analysis includes MozzareLLM results for both interphase (73 high-confidence clusters) and mitotic (10 high-confidence clusters) populations. It also opens to a default cell class and resolution, which is the MozzareLLM annotated one (can be manually set by user).

15. MozareLLM is a cool tool that could be useful beyond Brieflow -- mention it in the abstract so it can be more easily picked up in searches?

Thank you for the suggestion. We have now added a sentence to the abstract that states: "To accelerate biological interpretation, we additionally present MozzareLLM, a framework leveraging large language models to identify biological processes within phenotypic clusters and prioritize gene candidates for experimental validation."

16. I might have missed it, but is Ext. Figure 1 cited in the text? Maybe cite it in the first paragraph?

Thank you. We have added a citation to the figure to the first results section.

17. L. 45: "simplistic" sounds deprecating -- fitness-based screens can provide very useful insights. Use "simple" instead?

We have changed this to "simple" in the revised manuscript as suggested.

18. Fig. 1 might not be the most intuitive for non-experts. Consider adding more explicit visual details about the different steps of OPS (cf. Figure 1 in Funk et al?).

We have revised Figure 1 to include more explicit visual details about the OPS workflow, improving accessibility for non-expert readers.

19. L. 106: for non-experts, can you provide more intuitive context regarding the advantages of Snakefile, and why they are useful for Brieflow?

We have now added a statement to the results section to address this point that states: "The implementation leverages Snakemake-based workflow management, which automatically determines processing steps based on file dependencies for incremental updates, distributes independent tasks across available computational resources, and captures complete analytical provenance in a version-controlled format for reproducibility."

20. L. 158: "standard method based on statistical signal detection" - this requires more info, or at least a reference?

We have now provided additional information on this point stating that "a standard method based on classical image processing signal detection" with full algorithmic details in Methods describing the Laplacian-of-Gaussian filter, local maxima identification, and quality scoring approach.

21. The panel arrangement in Fig. 3 is a bit confusing. Consider re-organizing.

We have reorganized the panel arrangement in Figure 3 for improved clarity.

22. The "aggregation" aspect is not intuitively captured in Figure 3C. Consider adding two colored columns to the "Cells" matrix at the top: "Barcode" and "Genes", with multiple cells sharing the same gene. Then the "Perturbation" matrix at the bottom could be smaller and only show the "aggregated" genes with the same colors.

We have now revised the aggregation visualization to more intuitively capture the transition from single-cell measurements to perturbation-level profiles.

23. L. 212 what do you mean by "cellular objects"? How does the number 69,065,844 compare to the "70+ million cells identified" mentioned l. 236?

We have replaced "cellular objects" with explicit cell counts and clarified the relationship between module-specific counts. We now report SBS cells (74,406,684), Phenotype cells (71,629,095), and merged cells (54,683,953) with explanations that differences reflect distinct segmentation and filtering in each module. We have additionally added a visualization showing cell counts at each stage of the pipeline (Extended Data Fig. 5a).

24. L. 238: the "triangulation hashing" approach is not necessarily intuitive for non-experts. Consider adding give a more intuitive explanation re: what triangular hashing does. Which channels does it use?

We have now added additional information to the results to indicate that "Because OPS workflows typically image barcodes and phenotypes at different magnifications and potentially on different microscopes, direct pixel-to-pixel correspondence cannot be assumed. Instead, the merge process leverages the spatial pattern of cells themselves as fiducial markers: cells occupy consistent physical positions regardless of imaging magnification, so the geometric relationships between neighboring cells can be used to identify corresponding regions across modalities. The implementation uses Delaunay triangulation to create hash-based descriptors of local cell arrangements that can be matched across imaging modalities despite differences in scale and orientation."

25. Ll. 395-396: I got confused by the switch of naming nomenclature between C18orf21/NEPRO and RMP24/RMP64. Keep C18orf21/NEPRO nomenclature throughout to be consistent?

We thank the reviewer for this observation. In the revised manuscript, we have substantially restructured the case study analyses, and the naming inconsistency noted by the reviewer is no longer present.

26. Ext. Data Figure 9: why are there only 4 genes mentioned in cluster 60 in panel A, when more genes from that cluster are presented in panel B? Probably because panel A only covers only vesicular trafficking genes? The legend for panel A is a bit confusing, mentioning "The refinement reveals distinct vesicular transport modules including separated exocyst complex and CCR4-NOT components" which is not shown in that Figure.

We thank the reviewer for this feedback. The relevant Extended Data figures have been redesigned as part of the revised analysis and no longer contain the panels referenced by the reviewer.

Reviewer #3

1. The manuscript would benefit from an explicit statement of data-collection assumptions. For example, many instrument setups do not guarantee one-to-one tile correspondence across cycles; thus, the current solution may not suit all microscope configurations. A related issue is compatibility with the multiple-round phenotyping via iterative staining. Such limitations should be clearly stated to communicate the software's scope.

We thank the reviewer for this suggestion. For the revised manuscript, we have added the following information to the results section:

"The module supports multiple input formats through a configurable interface: users specify their file type (ND2 or TIFF) and associated metadata structure, enabling compatibility with diverse microscopy systems including Nikon (ND2), Phenix (TIFF), and Squid (TIFF) platforms... The module also supports multiple-round phenotyping via iterative staining, with each round treated as an independent phenotyping cycle that is processed separately before downstream integration. We note that the current implementation assumes one-to-one tile correspondence within phenotyping rounds and sequencing-by-synthesis cycles, which is satisfied by most standard microscope configurations."

2. The reanalysis of HeLa data should be evaluated and visualized more thoroughly. Embedding and clustering are sensitive to method choice and hyperparameters. For example, if Leiden clustering is applied to PHATE embeddings, varying the embedding dimensionality and other PHATE parameters will yield different clustering results (in particular, if clustering were performed on two-dimensional embeddings, substantial information would be discarded). While the Methods section describes evaluation against knowledgebases, please also assess embedding quality and present these results in the main figures.

(see point 3 below)

3. Similarly, many claims about improvement over the previous pipelines are not rigorously compared. While the current pipeline provides helpful case studies, it is also possible that the prior Vesuvius dataset captures other biological processes more effectively. A systematic, dataset-wide comparison demonstrating that embedding and clustering results are generally comparable or improved would help, before emphasizing improvements in specific clusters. If reanalyzing the Vesuvius dataset is not feasible, please temper and clearly qualify these claims.

We thank the reviewer for these two closely related comments, which motivated the most substantial additions to the manuscript.

For clustering evaluation, we now report quantitative benchmarking at multiple resolutions against three independent reference databases, including STRING protein-protein interactions, CORUM protein complexes, and KEGG pathways. For the systematic comparison with Funk et al., we applied MozzareLLM to both pipelines and performed comprehensive cross-pipeline analysis. As described in the results section, "Compared with the Funk et al. clustering at their optimal resolutions (k=10 interphase, k=9 mitotic; both producing 222 clusters), Brieflow's interphase clustering showed a higher STRING F1 score (0.098 vs. 0.067), CORUM enrichment (33.0% vs. 23.9%), and KEGG enrichment (18.1% vs. 13.5%)."

In total, we have added a new Results section with supporting Supplemental Tables S3–S8 to provide a robust dataset-wide comparison as suggested. This includes a balanced assessment showing that 35% of high-confidence Brieflow clusters (29 of 83) had no adequate match in the Funk clustering, compared with 21% of Funk clusters (14 of 68) that were reciprocally unmatched.

Together, we believe that this robust and quantitative comparison adds significantly to this paper and our ability to make clear points on the value of the Brieflow pipeline.

4. To support these points, consider merging the current Fig. 2 and Fig. 3 into a single figure, and dedicate one figure exclusively to QC/benchmarking on the reanalysis.

We have reorganized the figure layout to better present the benchmarking and reanalysis results.

5. Line 348 "a mean of 1.3 completely uncharacterized genes per cluster" reflects library composition rather than a property of MozzareLLM. Please adjust the wording of this section to avoid implying a discovery specific to MozzareLLM.

We have reworded this sentence to attribute gene composition to the clustering output rather than to MozzareLLM's analysis. This now states that "On average, high-confidence Brieflow clusters contained 16.6 genes with established functions in the

identified pathway, 7.4 genes with established functions but potential novel roles in the identified processes, and 1.7 genes with limited functional annotation — representing candidates that co-cluster with well-characterized pathway components (Extended Data Fig. 7c)."

6. line 345: When cluster labels are permuted, how many "High confidence" biological processes would be detected? I suggest adding such a baseline.

We have added this analysis as a shuffled negative control. As described in the results section, we now state:

"To establish that MozzareLLM pathway confidence scores reflect genuine biological signal rather than artifacts of the annotation framework, we generated a shuffled negative control by randomly permuting gene-to-cluster assignments within the Brieflow interphase dataset while preserving cluster size distributions. Running MozzareLLM on this permuted clustering yielded 256 clusters, of which only 1 (0.4%) received a high-confidence pathway annotation; that single cluster contained 6 genes, and subsequent inspection revealed it arose by chance from a random co-assignment of annotated paralogs. By contrast, the unshuffled Brieflow interphase clustering produced 73 high-confidence clusters (32.2%) containing 1,874 genes. The approximately 80-fold enrichment of high-confidence annotations in real versus shuffled data (32.2% vs. 0.4%) demonstrates that MozzareLLM's confidence assessment is not trivially satisfied by arbitrary gene groupings... Consistent with this, the shuffled clustering showed 0% CORUM and KEGG enrichment (compared to 33.0% and 18.1% for real interphase clusters) and a STRING F1 score of 0.003 (compared to 0.098), confirming that the biological signal captured by Brieflow's clustering is absent in randomized controls."

7. While the manuscript emphasizes modularity, there appears to be a learning curve for adding new functions to modules. I recommend organizing the repository and adding brief instructions wherever possible.

We have added brief instructions for extending individual modules, including guidance on how to create branches for custom implementations. Standardized interfaces between modules ensure that modifications to specific components can be implemented without disrupting the overall workflow.

8. Small test images downloadable from GitHub are very helpful for new users. Consider referencing them explicitly in the Data Availability section.

We have added a statement to the Data Availability that indicates: "A small test image dataset for validating Brieflow installation and testing pipeline functionality is included in the Brieflow repository at https://github.com/cheeseman-lab/brieflow/tests/small_test_analysis. This dataset provides representative examples of OPS images for both sequencing-by-synthesis and phenotyping outputs."

9. Extended Data Fig. 2 currently lacks illumination-correction results.

We have updated Extended Data Fig. 2 to include illumination-correction results showing the effect of correction on signal uniformity across the field of view.

The documentation was clear for installation and running the test that they prepared. I did not test it for real data.

We appreciate the reviewer's evaluation of the codebase. We have continued to improve the documentation, including comprehensive tutorial videos, detailed implementation instructions on readthedocs, and the small test dataset for pipeline validation.

We thank the reviewers for their supportive assessment of the revised manuscript and their helpful final suggestions. Below, we provide point-by-point responses to each comment.

Reviewer #2

Congratulations to the authors for the revised version of this manuscript. My comments have been successfully addressed during revisions, and I fully support publication.

We thank the reviewer for their thorough evaluation and support.

Review all Figure legends and maximize explicit explanations. It took me a while to understand some figure panels. Adding more info in the legend would be quite helpful. In some instances, the definition of some terms might not be intuitive to a general-audience reader. For example "mapped cells" in Figure 2C.

We have substantially expanded all figure legends to include explicit definitions of technical terms and panel descriptions. For example, in Fig. 2c, "mapped cells" is now defined as "the percentage of segmented cells for which a detected barcode was successfully matched to exactly one known sgRNA target gene." Similar clarifications have been added throughout all figure legends.

Fig. 3B "Representative snapshot of merge approach" graph - it took me a while to understand what you are trying to show in that panel. Consider extending the legend.

We have rewritten the Fig. 3b legend to provide a clearer description of all three sub-panels. The bottom-left scatter is now described as "representative snapshot of the merge process for a single tile, showing the overlay of phenotype cell positions (blue) and SBS cell positions (green) plotted in their respective per-tile coordinate systems, with gray lines connecting matched cell pairs across the two coordinate frames. Close spatial proximity of connected pairs confirms successful registration." The bottom-right panel is similarly clarified.

Fig. 3D: how were the specific principal components chosen? The figure seems to imply a correspondence between PCs and the original features such as mean DAPI intensity. If so, could you make this explicit in the legend and explain how it was determined? If not, and PCs were merely chosen to show normalization, consider saying that.

We have clarified this in the legend. The four raw features (left column) are representative features chosen to illustrate batch effects across common staining channels. The four principal components (right column) were randomly selected to show that batch-correlated variation extends beyond individual features into the PCA embedding space. The legend now explicitly states: "Left column: four representative raw features (mean cell DAPI, tubulin, γH2AX, and actin intensity). Right column: four randomly selected principal components (PCs 654, 114, 25, and 759), shown to illustrate that batch-correlated variation extends beyond individual features into the PCA embedding space."

l. 428 "MozzareLLM identified distinct high-confidence biological processes" -> how is "high confidence" defined?

The definition of confidence levels is provided in the Methods (MozzareLLM section): "high confidence requires greater than 70% of genes with strong literature support, medium confidence requires 50–70%, and low confidence requires 30–50%, with clusters below 30% designated as having no coherent pathway." We have ensured this definition is clearly referenced where high-confidence annotations are first introduced in the Results.

I had a hard time understanding what is shown in Fig. 5C and D. Be explicit about what each triangle of the heatmap represents, and label the axes? Is this showing the difference between PCA at the single-cell level vs after gene-level averaging? The corresponding part of the text could be clarified accordingly. The concepts of "raw interpretable features" (l. 497) or "PCA-based embeddings" (l. 500) are not very intuitive to understand.

We have substantially rewritten both the Fig. 5c/d legends and the corresponding main text to clarify what each panel shows. The two panels differ by feature representation type, not by pipeline: Fig. 5c shows a Pearson correlation matrix computed from PCA-based perturbation profiles, while Fig. 5d shows the same matrix computed from raw feature-based profiles. Within each panel, the upper triangle shows correlations from the Brieflow pipeline and the lower triangle shows correlations from the Funk et al. pipeline, enabling direct visual comparison of how each pipeline resolves mitochondrial sub-module structure under a given feature representation. The legend now explicitly defines each triangle and feature type. In the main text, we have added parenthetical definitions: "raw interpretable features (original measured features such as intensity and shape, aggregated per perturbation via median without dimensionality reduction)" and "PCA-based embeddings (single-cell feature vectors projected into principal component space, then aggregated

per perturbation via median)." We have also corrected the text to accurately state that aggregation uses the median (not mean) throughout.

Ensure that font size in all Figure panels is above a threshold. Some text is very small (e.g. axes labels).

We have reviewed all figure panels and increased font sizes where text was below the recommended threshold, particularly axis labels in Fig. 2c and gene labels in Fig. 5c/d.

Reviewer #2 (Remarks on code availability): I did inspect the code and found it well-documented and clearly organized. The Readthedocs documentation is helpful. I did not run the code on real data.

We appreciate the reviewer's evaluation of the codebase.

Reviewer #3

The authors have addressed my comments. I support publication in Nature Communications.

We thank the reviewer for their support.

Reviewer #3 (Remarks on code availability): I have installed and successfully ran their tests but have not applied to other datasets.

We appreciate the reviewer's evaluation of the codebase and successful execution of the test suite.
